# Use of Sensors and Analyzers Data for Load Forecasting: A Two Stage Approach

**DOI:** 10.3390/s20123524

**Published:** 2020-06-22

**Authors:** Daniel Ramos, Brigida Teixeira, Pedro Faria, Luis Gomes, Omid Abrishambaf, Zita Vale

**Affiliations:** 1 GECAD—Research Group on Intelligent Engineering and Computing for Advanced Innovation and Development, Rua DR. Antonio Bernardino de Almeida, 431, 4200-072 Porto, Portugal; dados@isep.ipp.pt (D.R.); bccta@isep.ipp.pt (B.T.); pnf@isep.ipp.pt (P.F.); lufog@isep.ipp.pt (L.G.); ombaf@isep.ipp.pt (O.A.); 2 Polytechnic of Porto, Rua DR. Antonio Bernardino de Almeida, 431, 4200-072 Porto, Portugal

**Keywords:** building energy management, demand response, load shifting, SCADA, user comfort

## Abstract

The increase in sensors in buildings and home automation bring potential information to improve buildings’ energy management. One promissory field is load forecasting, where the inclusion of other sensors’ data in addition to load consumption may improve the forecasting results. However, an adequate selection of sensor parameters to use as input to the load forecasting should be done. In this paper, a methodology is proposed that includes a two-stage approach to improve the use of sensor data for a specific building. As an innovation, in the first stage, the relevant sensor data is selected for each specific building, while in the second stage, the load forecast is updated according to the actual forecast error. When a certain error is reached, the forecasting algorithm (Artificial Neural Network or K-Nearest Neighbors) is trained with the most recent data instead of training the algorithm every time. Data collection is provided by a prototype of agent-based sensors developed by the authors in order to support the proposed methodology. In this case study, data over a period of six months with five-minute time intervals regarding eight types of sensors are used. These data have been adapted from an office building to illustrate the advantages of the proposed methodology.

## 1. Introduction

The electricity sector is facing several challenges due to concerns about environmental issues [1,2]. The efficient use of electricity can be improved with the support of smart grids [3]. In fact, in the context of smart grids, consumers can receive incentives for reducing electricity consumption in certain periods [4]. This is in the context of Demand Response (DR) programs, in which the consumers receive incentives or price signals in real-time. This enables them to modify consumption to reduce electricity costs [5].

For a building or facility, at the commercial, domestic, or industrial level, adequate planning of the targeted tasks and respective energy consumption forecasts is needed [6]. This is more feasible to improve their participation in DR programs. In the end, the available resources used will be optimized, and the energy bill will be reduced by adapting the consumption to the available opportunities in smart grids [7].

Focusing on building energy measurement, a real-time automatic energy forecast can be performed with data monitored in a building in the context of electricity to optimize energy management [8,9]. Different artificial intelligence techniques can be used [10,11,12,13]. Artificial Neural Networks (ANN) represent a model with neurons with weighted connections in a multilayer framework organized with an input layer, one or more hidden layers, and one output layer [14]. Another technique, K-Nearest Neighbors (KNN), maps data in the large resource space by adopting nonlinear mapping, developing linear regression in the resource space [15].

These algorithms were tested in other applications in the energy domain area including Short Time Load Forecasting (STLF). An ANN model is proposed with training on hourly data to forecast the electric load of the NEPOOL region, in reference [16]. KNN is stated as an effective algorithm in classification and regression problems and is tested for regression problems concerning energy prediction and superconductivity. The algorithm adapts the number of nearest neighbors to each data sample, reducing training complexity [17]. A new forecast model is suggested in reference [18], adding to the K-Nearest Neighbor the back-propagation present in the Artificial Neural Networks algorithm, forming the KNN-ANN model. This new model is compared with the original algorithm of KNN for a stock price prediction problem. During the event NPower Forecasting Challenge 2015, the BigDEAL team [19] proposed a methodology that uses several techniques, including ANN and RF, in order to predict daily energy usage of a group of customers. A system proposed in reference [20] evaluates the classification of students using the algorithms ANN and SVM, and analyzing the limitations of each method.

Energy efficiency in buildings is an interesting topic for all sectors of the power distribution network, from system operators to the end-users. Nowadays, energy efficiency becomes more evident than before, as lots of new concepts and technologies have been implemented in end-use buildings, such as Supervisory Control And Data Acquisition (SCADA) or IoT systems [21]. Using these new technologies enables all types of buildings, including residential, commercial, and industrial, to monitor and manage the data related to energy consumption. These data are considered as a fundamental basis for energy forecasting algorithms that have a direct effect on the electricity markets and policy formulations [22].

Most electricity consumers are good targets to forecast their daily energy consumption profile, leading them to have financial profits in their monthly electricity bills by reducing the excess and unused parts of energy consumption. Indeed, energy forecasting methods mainly focus on future load requirements that will be employed for infrastructure design and planning [23]. According to the review shown in reference [24], there are three main approaches for building energy consumption forecasting and modeling: physics-based, data-driven, and hybrid models. Each method has its own advantages and disadvantages; however, the data-driven method is shown as the most appropriate option to merge buildings in the smart grids. Energy forecasting in buildings is performed by relying on various types of sensors, and energy-related information mainly measured by smart meters [25,26]. Most of the forecasting models have not been validated through actual and technical data. Therefore, the need for validating forecasting models on implemented pilots, including all types of smart buildings, namely residential or office buildings, is evident in this context [27]. Regarding building measurement, as an example, focusing on electrical building measure, a real-time automatic energy forecast can be performed with data monitored in a building in the context of electricity, in order to optimize energy management [28].

In the present paper, the proposed methodology aims to provide a solution to improve the forecast of electricity consumption supported by different sensors (presence, temperature, consumption, humidity, etc.,) and forecasting algorithms. ANN [29] and KNN [30] are considered as decision-making approaches to be used in this paper. In the developed methodology, also described is how the system deals with the missing data. In the first stage of the proposed approach, the use of different sensors is discussed to decide which ones can contribute to improving the energy consumption forecasting of buildings.

In the second stage, the forecast service is run for each period of five minutes. If the obtained forecast error is greater than the defined threshold, the forecasting algorithm is updated for the training, using the most recently available data. Additionally, in this paper, a description of the sensor’s prototype developed by the authors is provided in order to support the sensor’s data collection.

After this introduction, the proposed method is described in Section 2, with details about each stage. In Section 3, the sensor’s prototype description is provided, showing all technical specifications of the system. A case study is described in Section 4 to validate and test the performance of the model, and its results are presented in Section 5. Finally, Section 6 presents the main conclusions of the work.

## 2. Methodology Description

**Figure 1:**
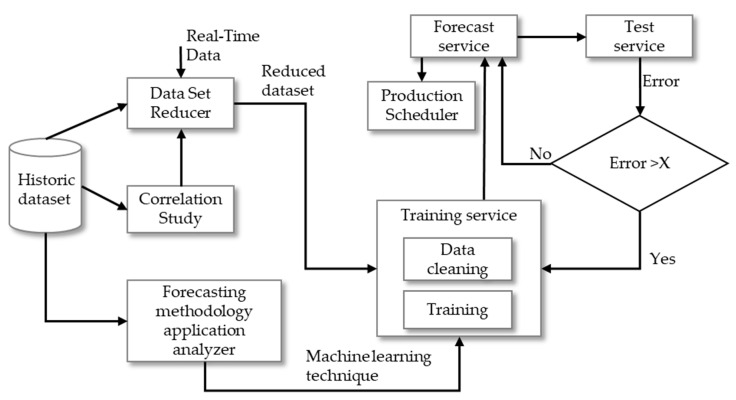
Proposed two-stage methodology diagram.

This section describes the different phases of the proposed methodology. This includes a definition of the running parameters, data import from the database, cleaning, training, import of the test parameters, forecast operations, presentation of the results, and their errors (see Figure 1). The steps of this two-stage approach are described in detail in Section 2.1, Section 2.2, Section 2.3, Section 2.4.

### 2.1. Tuning Process

The tuning process is a step that works exclusively for the parametrization of data involved in the data warehouse domain. The content involved in this field of study comprises two relevant aspects. The first is featured by a mechanism that evaluates the content of data in order to come up with the forecasting technique that is expected to provide better predictions in the specific case. The second one involves the creation of a replica of data with transformation changes that differ from the original version. These manipulations of data are set in action, evaluating the information more relevant that should be added to the data structure and discarding the remaining one.

There is a data structure balance between the simplicity of data to avoid wrong interpretability. It is also needed to maintain the completion of data to provide better predictions of future events. The content of the data determined through studies (in this step, it is significant to find the parameters) may result in forecasts with higher accuracy on future steps of forecasting. Moreover, it should be considered that the algorithm’s accuracy is highly dependent on the data structure and data reliability. In fact, this step is only performed once per execution of the algorithm.

The real-time data involves all the monitored, persistent and available data that the system keeps track of. The information corresponds to consumption and sensor data measured and monitored in the building. From all these data, a sample is selected to be studied and analyzed in the historic dataset. The correlation is applied in order to determine the most relevant sensors to be used. This is a relevant analysis that studies the strength of relation between the variables. This will determine the more relevant sensors to be included in the forecasting dataset. This study makes it possible to reduce the data, as the less relevant sensors are discarded from the data structure. However, in fact, it may happen that some sensors providing nonlinear behavior of the consumption data could help the forecasting algorithm to have better results. In this way, in the present paper, nonlinear relations between the consumption and sensors data are disregarded.

Although this reduction in data is applied to the historic dataset, the same rules obviously work for the whole content present in the real-time data. The new version of data with the reduced content is kept in the training service to be trained, but also in the forecasting service to perform prediction studies. Furthermore, a separate process known as the forecasting methodology application analyzer studies the historic dataset in order to determine which forecasting technique is expected to provide better insights. The forecasting technique is afterward sent to the training service. In this way, both the set of inputs and the forecasting technique that better fit the dataset under study are obtained.

### 2.2. Training Service

The training service can take action in the system in three ways: immediately after ending the tuning process operation; after the system receives a training request; after the error calculation requests a new retrain that checks if the results are not good enough. In the second case, it is important to guarantee that the tuning process has already defined the parameters.

The cleaning operation reorganizes the data in a structure to make it more suitable for the train/test split, considering several factors. First of all, all the data are reorganized in a unique spreadsheet with a date split into several fields (year, month, day of the month, day of week, hours, and minutes). Moreover, missing information is added following a criterion which makes sequential copies from previous records. Additionally, information associated with weekdays is considered unreliable and as a matter of fact, records following this assumption are excluded in the spreadsheet. Afterward, an automatic algorithm is applied to deal with the outlier issue. This occurs due to erroneous readings made by the devices that measure the consumption and sensor data. The algorithm’s strategy consists of detecting occurrences with variations outside of the normal in the dataset. This is done with the support of the mean and standard deviation operations. For each value in the dataset, the mean and standard deviation are calculated for a particular distribution with the actual record and a limited number of records that occurred previously and afterward. The mean and standard deviation are calculated respectively in Equations 1 and 2. These calculations are simple and can be made using a spreadsheet.

(1) $$A=\frac{\sum_{t=n-F}^{n}P\left(t\right)}{F}$$

- *A*—average consumption in *F*;
- *n*—current moment;
- *P*—consumption;
- *t*—period;
- *F*—frame used for calculation.

(2) $$S=\sqrt{\frac{1}{F}*\overset{n}{\underset{t=n-F}{\sum }}{\left(P\left(t\right)-A\right)}^{2}}$$

- *S*—standard deviation consumption in *F*;
- *F*—frame used for calculation.

Every time, that the actual value is lower or equal to the mean minus the error factor times standard deviation, the actual value is replaced by the mean of the previous and following records (see Equation (3)). This is also true while the actual value is greater or equal to the mean plus the error factor times standard deviation. The error factor is defined during the tuning process according to the experimenting of different values. A common value is 2.

(3) $$P\left(n\right)\geq A+\varepsilon S\;˅\;P\left(n\right)\leq A-\varepsilon S\;∶=\;P\left(n\right)=\frac{P\left(n-1\right)+P\left(n+1\right)}{2}$$

- *ε*—error factor.

Finally, as the service name indicates, the system extracts a historic from the cleaning data to be kept with a suitable structure. This is used by the forecasting technique (the same structure defined in the tuning process).

### 2.3. Forecasting Service

The forecasting service can take action in the system in three ways: immediately after ending the training service operation; right after the system receives a test request; or even right after performing a new iteration after ending the error calculation process synced with the scheduled activity. In the second case, it is important to guarantee that the training process has already performed its tasks. This process can be done several times in the algorithm.

The forecasting service starts to read test parameters associated with the iteration, which syncs the information with the time schedule. The fields presented in the test dataset are the input data and the respective target that the program is supposed to forecast.

Afterward, the forecasts are scheduled to be performed in a specific period for the target defined previously with the technique determined in the tuning service. The forecast values are targets representing total consumption.

### 2.4. Test Service

The test service step takes action instantly after the forecasting service ends its tasks. As the service name indicates, the forecast error is calculated for the moment which will determine how far the forecast value is from the actual value.

The errors are calculated based on three possible metrics: Weight Absolute Percentage Error (WAPE), Square Mean Absolute Percentage Error (SMAPE) and Root Mean Square Percentage Error (RMSPE) (Equations (4)–(6)).

(4) $$WAPE=\frac{\sum_{t=n-F}^{n}\left|P\left(t\right)-PF\left(t\right)\right|}{\sum_{t=1}^{n}P\left(t\right)}$$

(5) $$SMAPE=\frac{1}{F}*\overset{n}{\underset{t=n-F}{\sum }}\frac{\left|PF\left(t\right)-P\left(t\right)\right|}{\left(P\left(t\right)+PF\left(t\right)\right)/2}$$

(6) $$RMSPE=\sqrt{\frac{\sum_{t=n-F}^{n}{\left(\frac{P\left(t\right)-PF\left(t\right)}{P\left(t\right)}\right)}^{2}}{F}}$$

- *PF*—forecast consumption;
- *F*—frame used for calculation;
- *t*—period.

Following this, a condition is tested in order to check if the error associated with the moment in question is acceptable—in other words, if it is low enough according to a trigger criterion. If the error is not low enough, the trigger is activated, which automatically sends a new train request in order to rerun the train service. However, the implicit train will be a new train with updated content composed of information contained in the test service that was previously rerun just before the trigger point. The new historic size is kept exactly as the old counterpart and while the historic is updated with new content, the old content is discarded. The test will be associated with the information from the trigger point until the end of the week. If the error is low enough, there are two possible alternatives. The error calculation is repeated for each new iteration or the loop is broken, which will end the entire process.

## 3. Sensor Prototypes

The proposed methodology uses data provided by sensors developed by the authors. These sensors are installed inside the building to give precious data for the methodology. Therefore, the sensors enable the consumption forecast algorithm to consider the building’s context. The used sensors are part of a multi-agent system where each sensor is connected to an ATmega328-P microcontroller and a nRF24L01 module. This enables the autonomy of each sensor. The sensors/agents are self-triggered using the internal clock, enabling the sending of real-time data through the radio frequency module. The sensors are also capable of learning rules, pursuing goals and reacting to the environment. However, these functionalities will not be used in the proposed methodology. The sensors are used to monitor real-time data and to store these data to be later used in the training service.

The sensor’s multi-agent system uses a compound organization with a combination of federative, team and congregation organizations. The system can have two types of agents: common agents, and delegate agents. Common agents with the same sensor type (i.e., movement) in the same room form a congregation. All sensors in the same room form a team. All sensors in the building form a federation, where a delegate agent represents the entire system in an Internet of Things (IoT)-based architecture. The delegate agent does not have any sensor, and it is used as a gateway between radiofrequency messages and Message Queuing Telemetry Transport (MQTT) messages. The delegate agent is also responsible to route the MQTT messages to the common agents.

**Figure 2:**
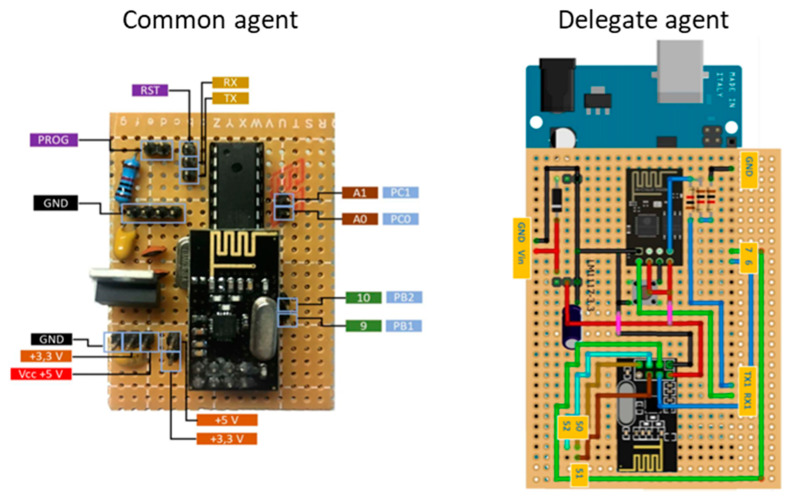
Agent types used in the sensor’s multi-agent system.

Figure 2 shows the two possible hardware boards for: common agents, and delegate agents. Each common agent integrates a single sensor, of any type. To allow MQTT messages, delegate agents use an Arduino Mega 2560 Rev3 and an ESP8266-01.

Because it is a federative organization, external systems only interact and see the delegate agent. Therefore, all the data are queried to the delegate agent, which in turn, will query common agents in order to make the data reply. The building’s SCADA system queries the delegated agent every five minutes in order to acquire real-time data. All these data are then stored in a database.

The proposed methodology also needs energy data regarding building consumption. These data are monitored using energy analyzers installed on electrical boards. The building’s SCADA system is responsible for querying, using Modbus/RTU, all energy analyzers and stores their data every five minutes. Besides the mentioned data, the SCADA system is also responsible for monitoring and storing the data of photovoltaic (PV) generation. The generated data are monitored directly from the PV inverter using Modbus/RTU protocol.

## 4. Case Study

The historic data of the building selected for this case study are divided into three different zones and are provided in five-minute time intervals. The selected dataset is from 22 May 2017 to 15 November 2019. Each zone has three rooms which include PV generation and consumption of loads (total energy consumption and power), and sensors data. This case study focuses on the sensors of zone 1, including:

- Four movement sensors;
- Three door status indicators;
- One air quality sensor;
- One temperature sensor;
- One humidity sensor;
- One CO_2_ sensor;
- Seven light power indicators.

**Figure 3:**
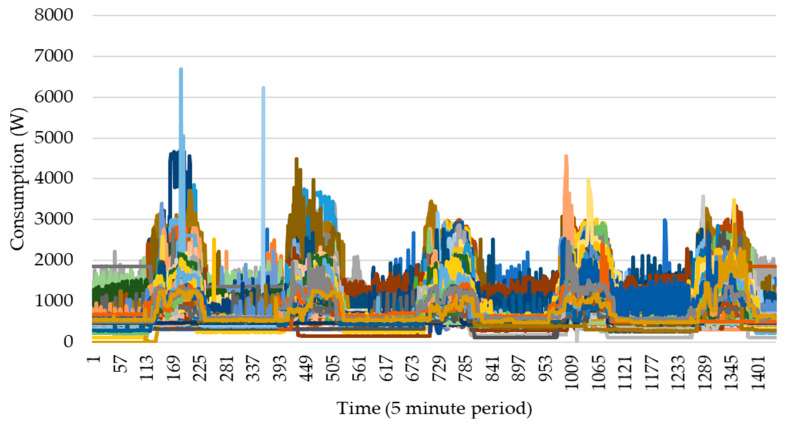
Weekly consumption profiles from 22 May 2017 to 15 November 2019. Each line is one week with 1440 points.

Outside of the rooms, the data in the corridors are monitored as well, which include the light power and the total consumption. The weekly consumption profile can be seen in Figure 3, for all weeks in the dataset. In this way, each series in Figure 3 (colors have no special meaning as these are any week profiles) have 1440 points in time axis which corresponds to one week of five days with five-minute intervals.

**Figure 4:**
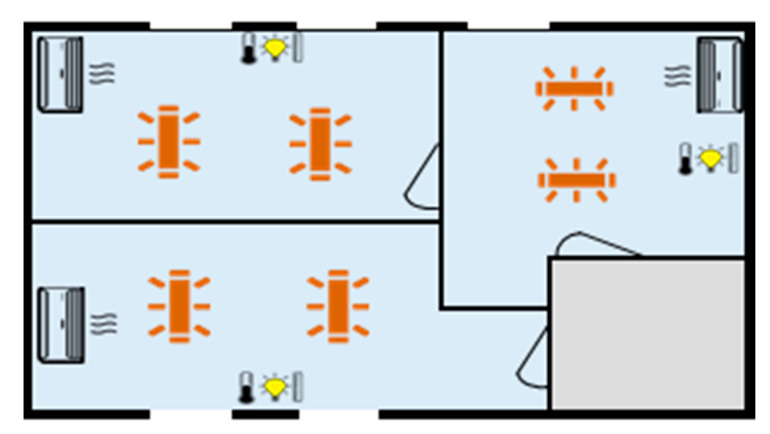
Plan of the building, consumption loads and installed sensors.

Figure 4 illustrates the plan of the building and the controllable devices in each office room. Due to space limitations in this paper, the sensor’s data are not presented. These data are also available with five-minute time intervals, similar to the consumption data shown in Figure 3. The plan of the building is composed of temperature and light sensors, as well as controllable loads (air conditioning devices and lights).

## 5. Results

This section presents the obtained results of the proposed methodology applied to the case study presented in Section 4. In Section 5.1, Section 5.2, Section 5.3, Section 5.4, the results are presented for the different phases of the proposed methodology.

### 5.1. Tuning

The tuning observations follow the step methodology illustrated in Figure 1 and detailed in Section 2.1. The first step aims to understand which foresting technique is more accurate to perform forecast consumptions targeted for specific data. A scenario is proposed using nearly two and a half years historic data (from 22 May 2017 to 8 November 2019) targeted for all five-minute time intervals. The data belong to 11 November 2019 until 17 November 2019. The goal is to use data in order to predict the consumption placed in the targeted period and focus on the mentioned area. The data include consumption and sensor information that measures temperature, humidity and light intensity from this area. The input data to the ANN and KNN results in the two right columns in Table 1 has been defined using the historic data of the sensors selected from the results shown in Table 2. The set of algorithms that test this scenario includes ANN and KNN. Three metrics are calculated based on the presented scenario for both algorithms, as can be seen in Table 1.

The presented errors show that in the ANN approach, the error is lower. This means that it can be considered as the most accurate technique. Therefore, employing ANN as the definitive forecasting technique was decided.

The sensor data used can also have a great impact on the forecasts. To understand which sensor data are more relevant to be included, the correlation matrix is built for each data column, as can be seen in Table 2. It should be noted that light consumption in this paper refers to the electricity consumption of the bulbs installed in the building. However, light intensity includes the level of illumination of the environment, which depends on the building’s bulbs as well as the available natural light.

The correlation values show that light intensity and CO_2_ sensors are more reliable due to their high correlation strength. Therefore, they have been selected to be used and surveyed in this paper.

Regarding the structure of ANN, it consists of a feed forward network representing a multilayer model composed of neurons and weights linked together. This structure is featured by one input layer with 10 neurons, followed by two hidden layers of 64 neurons each. This ends in an output layer with a single output. The amount of neurons in the input layer holds an extent of 10 consumptions placed in sequential periods. The output layer has only one value, which is the consumption that takes place after the last input. Furthermore, the epochs of ANN were defined with 500 iterations, which means ANN will keep training the model 500 times. The learning function used in the training process was the gradient descent algorithm. The learning rate was defined with 0.001, a very small rate considering the necessity to reduce the loss of information.

Regarding the KNN, the assumed set of parameters were: the k-nearest neighbor algorithm is associated with the five nearest neighbors; the weight function used in prediction is the uniform function, which states that all points in each neighborhood are weighted equally; the program is configured to decide the best algorithm option used to compute the nearest neighbor—the most adequate one based on the training and test data provided; the leaf size passed to the algorithm is 30; the distance metrics used for the tree is Minkowski; the power parameter used for the Minkowski metric is 2, which is equivalent to the Euclidean distance; the number of parallel rods to run for the neighbors search is one.

### 5.2. Data Cleaning

Data cleaning is made according to the methodology illustrated in Figure 1 and detailed in Section 2. Different types of data cleaning operations were performed in order to evaluate the more accurate ones. Missing information occurrences through the entire dataset are an issue due to lack of historic observations. The provided solution for this data cleaning operation consists of adding missing information for the problem at hand. The strategy consists of replicating the previous iteration value to the missing record.

**Figure 5:**
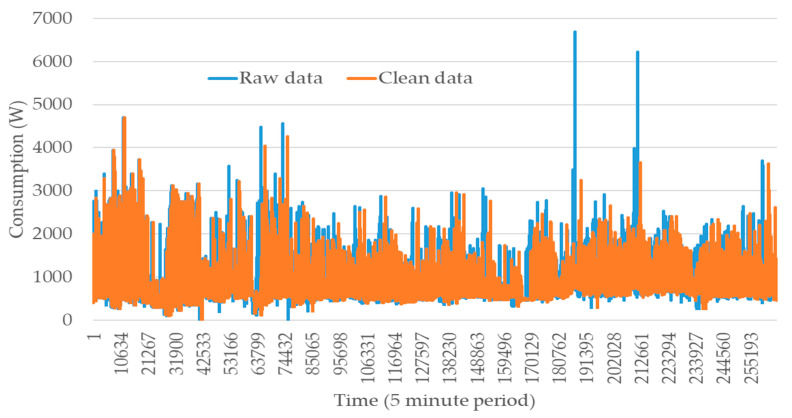
Consumption profile from 22 May 2017 to 30 November 2019 before and after cleaning.

Other issues that downgrade the forecasting performance are associated with the spikes and outlier’s existence. However, while it is relevant to discard outliers due to its erroneous data, the remaining spikes may be relevant to be aware of in some case scenarios. Therefore, only spikes representing outliers should be discarded from the historic data. In order to understand the data’s impact, two different versions of the consumption dataset are compared: one for raw data, and the other one for the results of data transformations that include missing information handling and the removal of outliers. This is shown in Figure 5.

The dataset size with treatment adjustments is longer than the raw data version as its cleaning operations include the addition of missing data, which increases the dataset size. Therefore, a lot of patterns presented in the original version and cleaned dataset are identical in different periods. Furthermore, the outlier existence is erased in the final dataset. The removal of outliers has a huge impact on the data as it corrects a lot of erroneous errors, making the consumption progress more accurate. This is verified in almost all periods, with lower or higher impact. While most corrections have a small impact, it can be verified that some exceptions contribute to a high-level improvement. These can be observed:

- In the period [19,453–25,937], replacing a consumption higher than 2000 W with one below 1000 W;
- In [51,873–58,357], replacing a consumption higher than 3000 W with one below 3000 W, deleting the two highest consumptions;
- In [181,553–213,973], exceeding 6000 W and replacing them with values between 3000 and 4000 W.

In the present case study, the value of error factor used in Equation (3) was 2.

### 5.3. Training and Forecast Dataset

**Figure 6:**
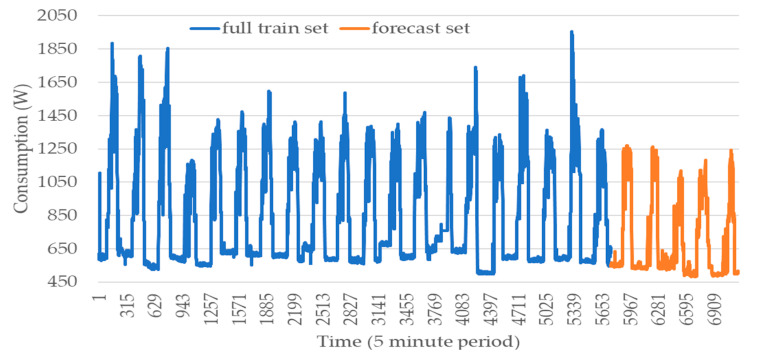
Consumption monitored from 11 to 15 November 2019 and historic with the 20 working days that precede 11 November 2019.

The training and forecast datasets are integrated into the methodology illustrated in Figure 1 and detailed in Section 2. The obtained results of this scenario focus on studying the information that will be considered for the training and test sets. The test set intends to use all five-minute records from 11 November 2019 to 15 November 2019. The historic information is composed of the 20 working days (excluding weekends and holidays) that precede 11 November 2019. Figure 6 presents the consumption during the train and forecast sets.

The daily consumption variability keeps a similar pattern in almost every scenario, despite the consumption differences in each day. As can be seen in Figure 6, the consumption profile switches multiple times between high increases and decreases. The nonlinear effect has been represented by a high increase in consumption. This is followed by a high decrease that represents all the five instance consumption measures framed in a day. The consumption range measured during the day varies from nearly 450 to 1900 W. The consumption behavior gains activity in the morning, taking measures with a minimum of 800 W and a maximum of 1900 W. This variation between 800 and 1900 W depends on the schedule of the machine. This behavior loses activity in the evening, taking consumptions between 450 and 650 W. The consumption activity range is very different from day to day but also from week to week. There are cases where this activity reaches consumptions from 1650 to 1900 W including the first, second and third days of the first week, the last day of the third week and the second and fourth day of the last week of training. Furthermore, there are a lot of cases where the consumption does not exceed 1400 W and a few where the consumption does not reach 1250 W during the entire day.

### 5.4. Forecast

**Figure 7:**
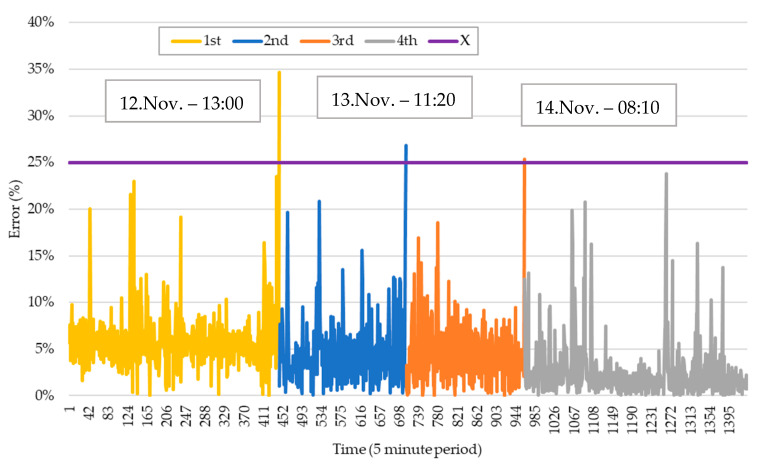
Forecasts errors from 11 to 15 November 2019: with the 1st, 2nd, 3rd, and 4th train.

This section proposes the results of the forecasts performed for all five-minute records shown in Section 5.3. As was mentioned in Section 5.3, the real consumptions for each moment of the test are monitored. Therefore, it is possible to calculate the errors associated with each moment. This procedure was illustrated in Figure 1 and its metrics calculation is explained in this section. This will provide insights about how accurate the forecasts are for each particular time interval. However, there are exceptions where the error is not low enough, which means the forecast is not accurate enough. To deal with this issue, it is important to define in the test set an error trigger of 25%. This means every time that the moment error exceeds the limit, the system should redo the forecasts using a historic one with updated content and a fixed size of 20 days. The training set will discard some of the initial records in order to keep a fixed size of 20 working days while adding updated content. This updated content will go just until the occurrence of the error trigger. The new version of the test set is composed of the remaining working days, counting only from the period to where the error trigger is activated. The unit used for the moment error will be the first one found. Figure 7 presents all the moment errors for all training and retraining. In this part, the error threshold (X parameter in Figure 1) has been set to 25%. This means that if the error is bigger than 25%, the forecasting algorithm is trained again with the most recent data.

The error trigger of 25% is activated on periods 445, 713 and 963. The error is shown by a nonlinear behavior that keeps increasing and decreasing gradually. While the first train keeps the forecast error under control, a second train with updated content is required for a new test starting in period 445. Afterward, the forecast error is once more kept under control until period 713. Therefore, the error trigger will be activated and ask for a new train with updated content and a new test starting from the period where the anomaly was detected. The same effect applies to period 963. From this point, the error is initially maintained under control and keeps its variations. This has been verified in the period [1101–1251], in which the maximum and minimum forecast errors will keep almost the same value. Afterward, the error is again out of control until the end of the test.

**Figure 8:**
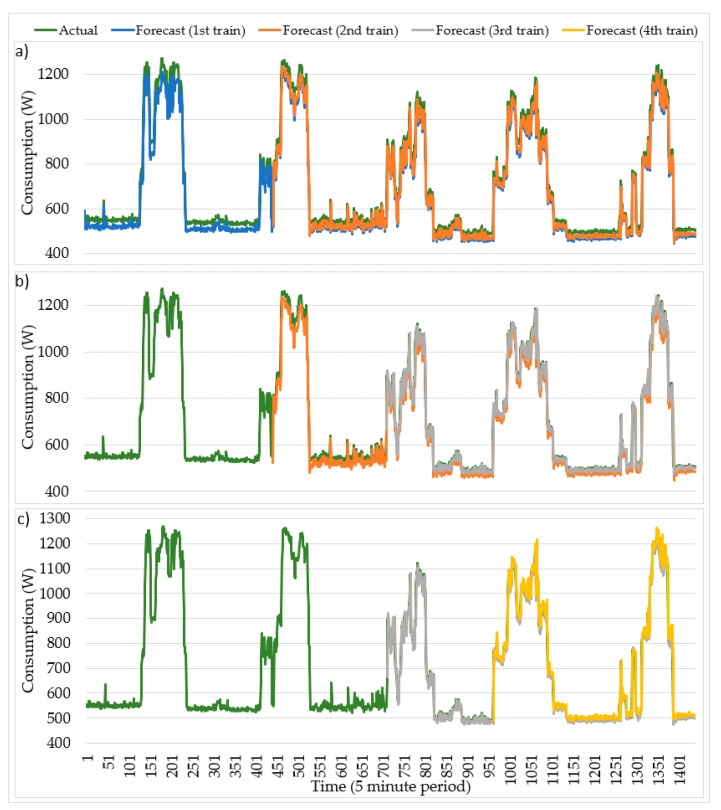
Consumption forecast from 11 to 15 November 2019. (**a**) Results of the first and second trains; (**b**) Results of the second and third trains; (**c**) Results of the third and fourth trains.

The real consumption, forecast and reforecast profiles from 11 November to 15 November 2019 are illustrated in Figure 8. The first train follows a similar pattern to its real counterpart during the whole process. There is a small consumption difference in the periods [0–130] and [220–400]. Furthermore, there is a local minimum in the period [130–173] which has low forecast accuracy. Following this occurrence, highlighting two patterns, a gradually increasing and decreasing consumption has small consumption differences.

The second train tends to follow a similar pattern to its real counterpart during the whole process. This is, however, a small consumption difference in the period [460–510], which is complemented by a low forecast accuracy of a local minimum and a local maximum. The third train and fourth train tend to follow a similar pattern to their real counterparts. The final forecast errors for each train are calculated and shown in Table 3. In Figure 8, each training method has been labeled with a different color. The illustrated data in the same figure are for a week with five working days in five-minute time intervals (1440 periods in total). More specifically, the results of the first and second trains are shown in Figure 8a, the second and third trains in Figure 8b, and finally, the results of the third and fourth trains are demonstrated in Figure 8c.

As is clear in Figure 8, except for the first day, the actual profiles of the other four days have been forecasted by more than one train. This leads to the conclusion that using trains with updated content is more accurate for the forecast. Again, Figure 8 has 1440 points in the time axis which corresponds to one week of five days, with each day represented by 24 h with 12 periods of five minutes each.

The progress from the first train to the second train and then to the third train with updated content clearly shows an improvement change based on the error analysis. However, the forecast for the fourth train is worse than the third, and better than the second approach, due to less reliability at the end of the week.

## 6. Conclusions

This paper proposes an automatic energy consumption forecasting for a building equipped with various types of sensors and energy monitoring equipment. The forecast method aimed at a set of five-minute time intervals and are supported by two algorithms (Artificial Neural Networks and K-Nearest Neighbors) using the Python language.

After applying the developed cleaning operation, only the more relevant data are used as input to the forecast algorithms. Graph visualizations show that this step leads to more accurate forecasts. Furthermore, the system performs forecasts on two independent processes storing all the results. The outcomes include the real and forecast test consumptions and respective errors associated with each moment and to the whole period. The first process’ forecasts were validated based on Artificial Neural Networks and K-Nearest Neighbors techniques and based on different scenarios with and without sensor data. The results of the paper demonstrated that the Artificial Neural Networks algorithm with sensor data has more accurate information. This is supported by the presentation of forecast result period errors and variables correlation study. It is important to highlight that the main results are only obtained at the end of the second process. Following this, the results obtained in the first process limit the number of test scenarios for the second process. In other words, there is a reduced number of tests in order to obtain the main outcomes. The results in this final process were shown by the graph moment forecasts and the period errors. From these results, it can be concluded that each retrain has advantageous implications in the forecast. All the simulations provided results with error between 4% and 7%, which are generally lower for each retrain with updated content, as proposed in the developed methodology.

As future work, multilayer models and more algorithm implementation should be done in order to perform more forecast tests that might achieve more accurate forecasts.

## Figures and Tables

**Table 1:** Forecast result errors with different methods and dataset.

Data	Using Consumption Data		Using Consumption and Sensor Data	
Method	ANN	KNN	ANN	KNN
WAPE (%)	2.44	2.81	2.38	3.52
SMAPE (%)	2.29	2.54	2.24	3.35
RMSPE (%)	4.11	4.92	4.17	6.28

**Table 2:** Correlation matrix of consumption and sensor data.

	Correlation							
Total consumption	1							
Total PV	0.3374	1						
Light intensity	0.8484	0.3681	1					
CO_2_	0.5403	0.4918	0.5677	1				
Air Quality	−0.2064	−0.3193	−0.2665	−0.2831	1			
Temperature	0.4178	−0.0132	0.2487	−0.0037	0.2205	1		
Humidity	0.0679	0.044	−0.0305	−0.0377	0.2348	0.296	1	
Light consumption	0.3376	0.2738	0.468	0.4154	−0.2326	−0.0568	−0.2516	1

**Table 3:** Forecast errors in the proposed methodology.

	Historic	Forecast	Trigger	ANN	WAPE	SMAPE	RMSPE
1st train	9-10 to 8-11	11-11 to 15-11	0	ANN1	6.02%	6.20%	6.95%
				ANN1 [11-11 to 15-11]+ANN2 [–]	6.02%	6.20%	6.95%
2nd train	10-10 13:00 to 12-11 12:55	12-11 13:00 to 15-11	445	ANN2	6.02%	6.20%	6.95%
				ANN2 [11-11 to 12-11 12:55] + ANN3 [12-11 13:00 to 15-11]	5.02%	5.13%	6.19%
3rd train	11-10 11:20 to 13-11 11:15	13-11 11:20 to 15-11	713	ANN3	5.02%	5.13%	6.19%
				ANN3 [11-11 to 13-11 11:15] + ANN4 [13-11 11:20 to 15-11]	4.12%	4.13%	5.72%
4th train	14-10 08:10 to 14-11 08:05	14-11 08:10 to 15-11	963	ANN4	5.02%	5.13%	6.19%
				ANN4 [11-11 to 14-11 08:05] + ANN5 [14-11 08:10 to 15-11]	4.47%	4.51%	5.95%

## Nomenclature

*n*	Current moment
*t*	Period
*A*	Average consumption in F
*S*	Standard deviation consumption in F
*F*	Frame used for calculation
*P*	Consumption
*PF*	Forecast consumption
*X*	Trigger error
*ε*	Error factor
